# Supplementary material for: Has COVID-19 Affected DTP3 Vaccination in the Americas?
Source: Vaccines (Basel). 2024 Feb 25;12(3):238. doi: 10.3390/vaccines12030238 (PMC10975091; doi:10.3390/vaccines12030238)
Supplement: Supplementary file 1 [file vaccines-12-00238-s001.zip › Suplementary Figures.pdf]

SUPPLEMENTARY MATERIAL

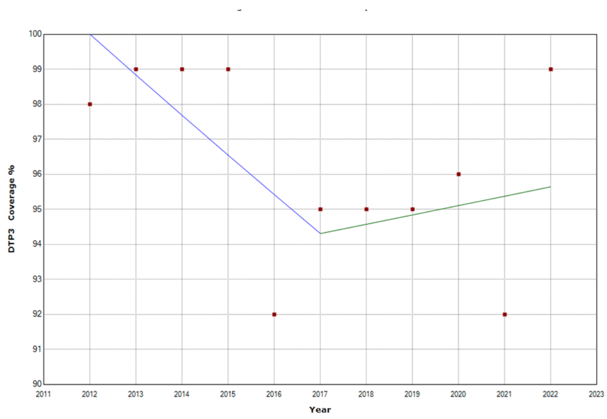

Figure S1. Joinpoint graph of DTP3 in Antigua and Barbuda (2012-22).

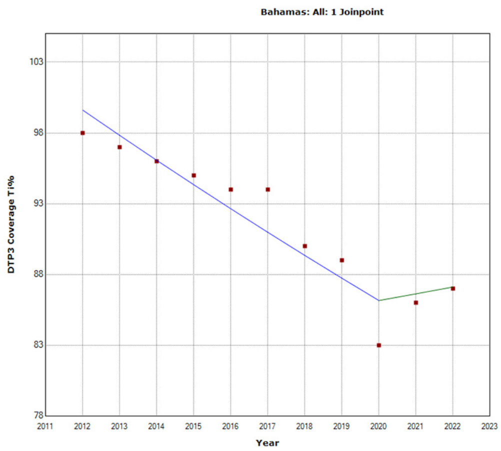

Figure S2. Joinpoint graph of DTP3 in the Bahamas. (2012-22).

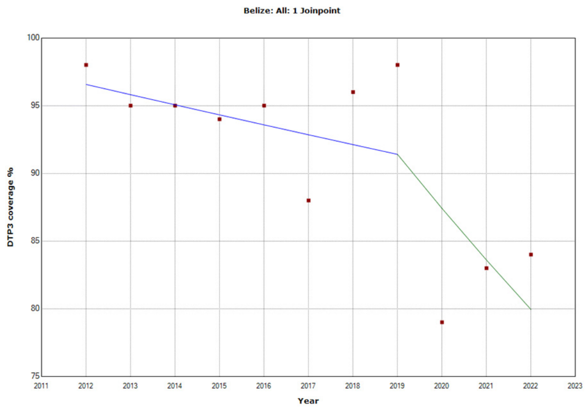

Figure S3. Joinpoint graph of DTP3 in Belize. (2012-22).

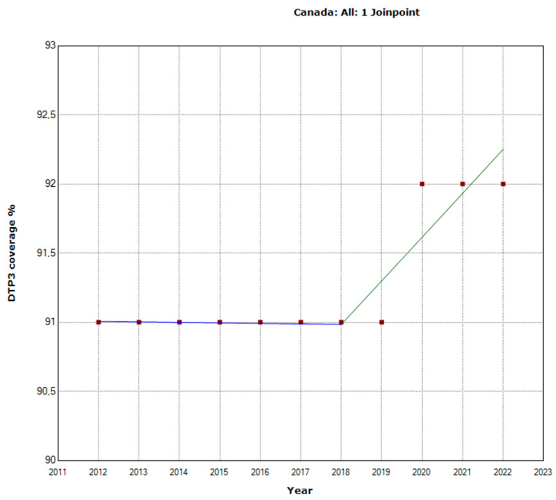

Figure S4. Joinpoint graph of DTP3 in Canada (2012-22).

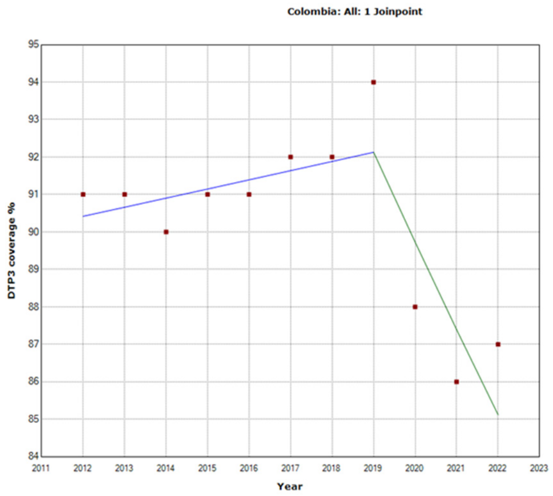

Figure S5 Joinpoint graph of DTP3 in Colombia (2012-22).

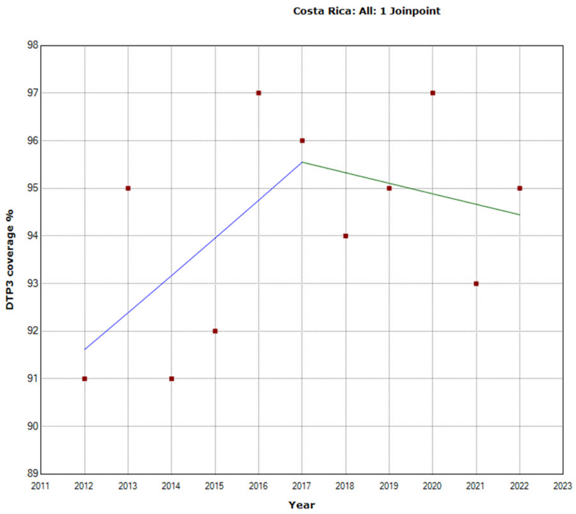

Figure S6. Joinpoint graph of DTP3 in Costa Rica (2012-22).

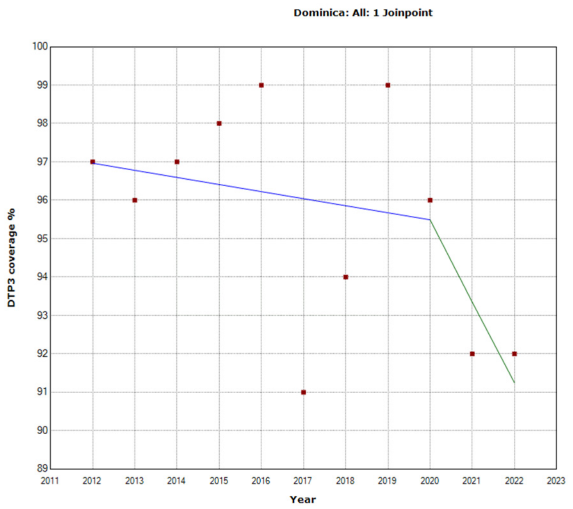

Figure S7. Joinpoint graph of DTP3 in Dominica (2012-22).

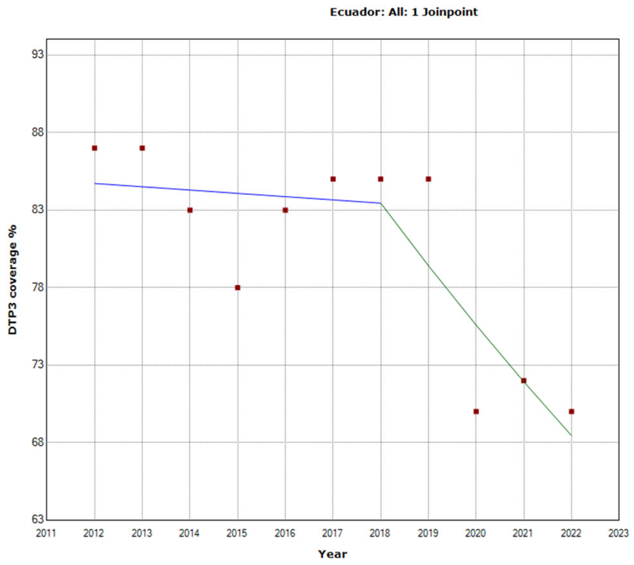

Figure S8. Joinpoint graph of DTP3 in Ecuador (2012-22).

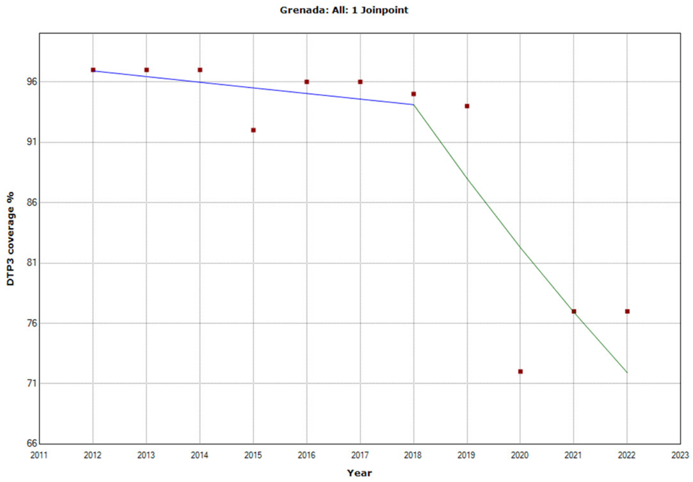

Figure S9. Joinpoint graph of DTP3 in Grenada (2012-22).

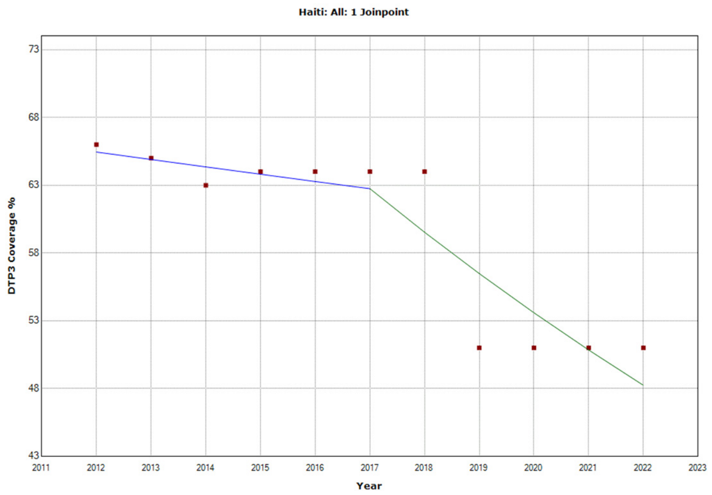

Figure S10. Joinpoint graph of DTP3 in Haiti (2012-22).

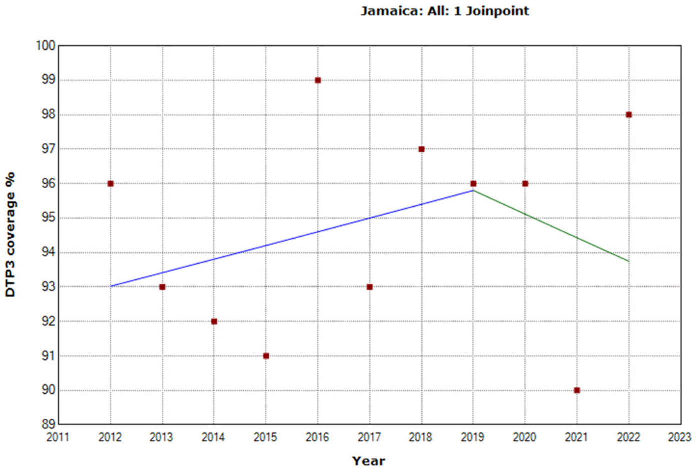

Figure S11. Joinpoint graph of DTP3 in Jamaica (2012-22).

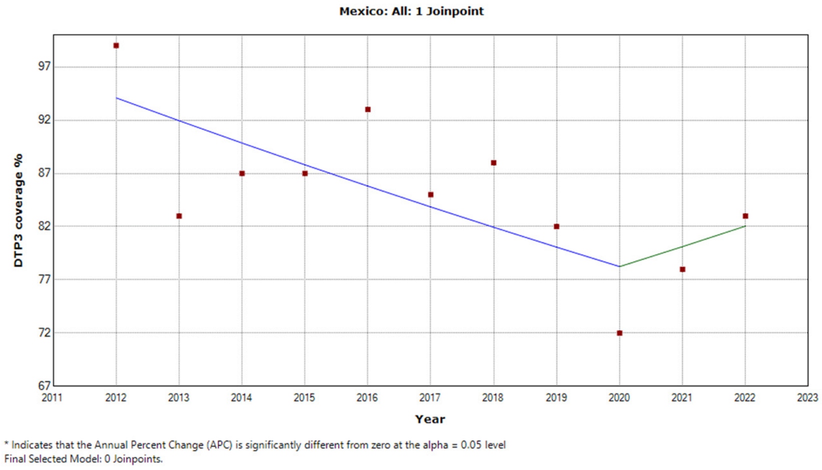

Figure S12. Joinpoint graph of DTP3 in Mexico. (2012-22).

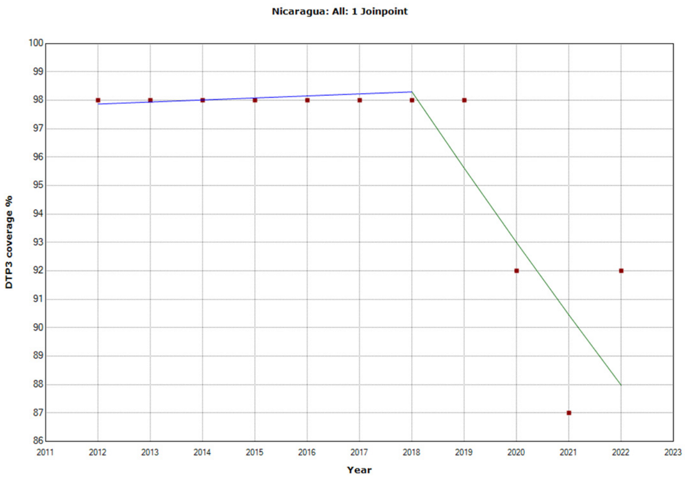

Figure S13. Joinpoint graph of DTP3 in Nicaragua (2012-22).

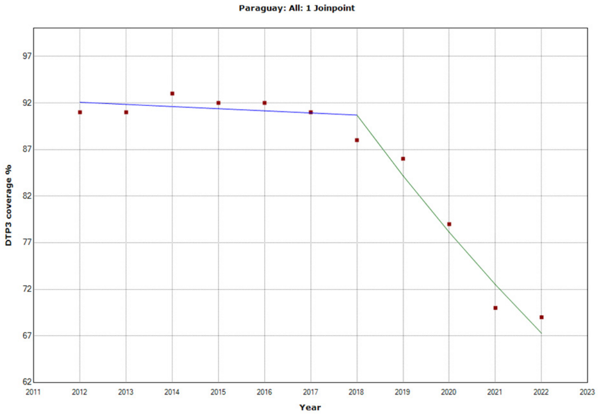

Figure S14. Joinpoint graph of DTP3 in Paraguay (2012-22).

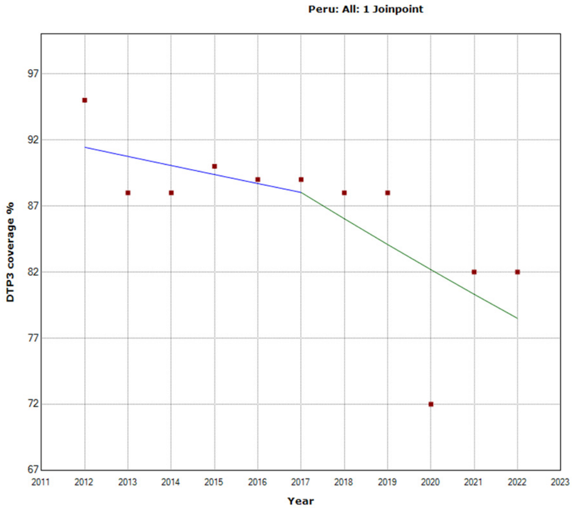

Figure S15. Joinpoint graph of DTP3 in Peru (2012-22).

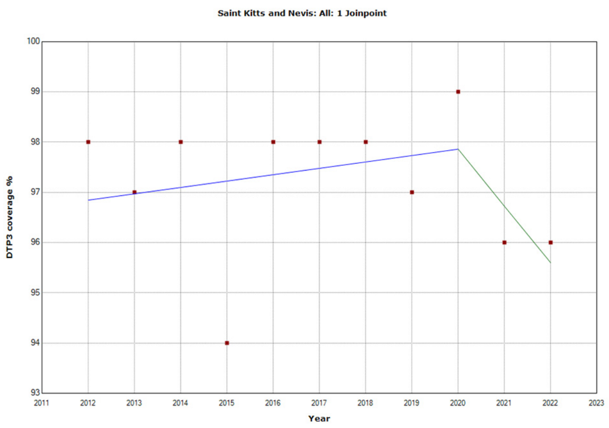

Figure S16. Joinpoint graph of DTP3 in Saint Kitts and Nevis (2012-22).

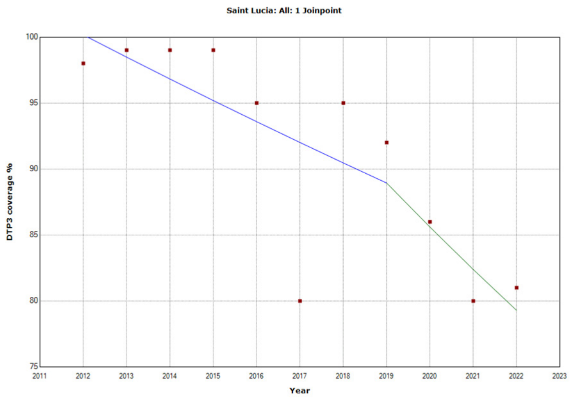

Figure S17. Joinpoint graph of DTP3 in Saint Lucia (2012-22).

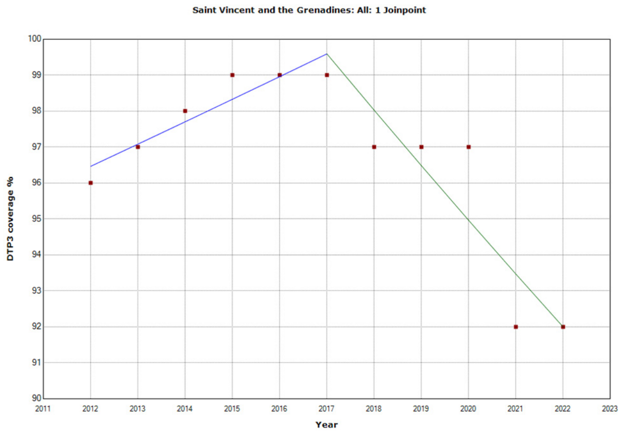

Figure S18 Joinpoint graph of DTP3 in Saint Vincent and the Grenadines (2012-22).

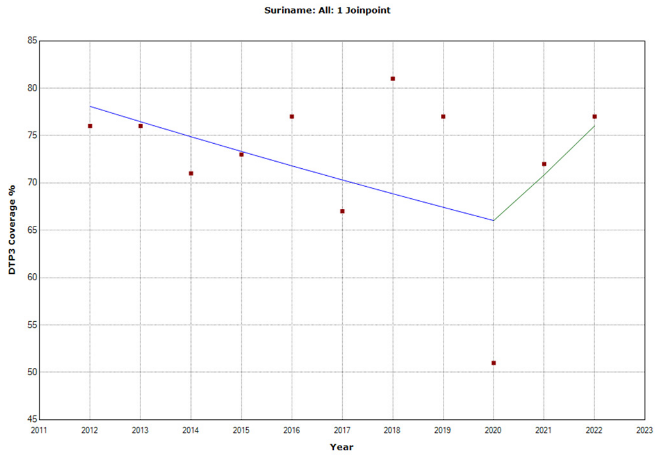

Figure S19. Joinpoint graph of DTP3 in Suriname (2012-22).

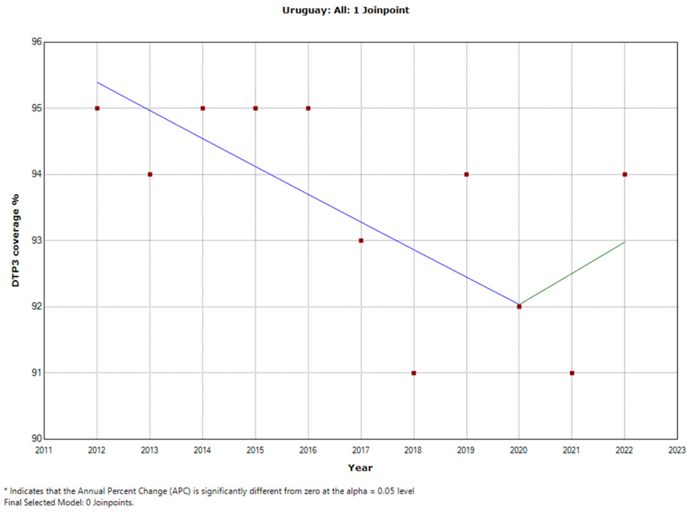

Figure S20. Joinpoint graph of DTP3 in Uruguay (2012-22).

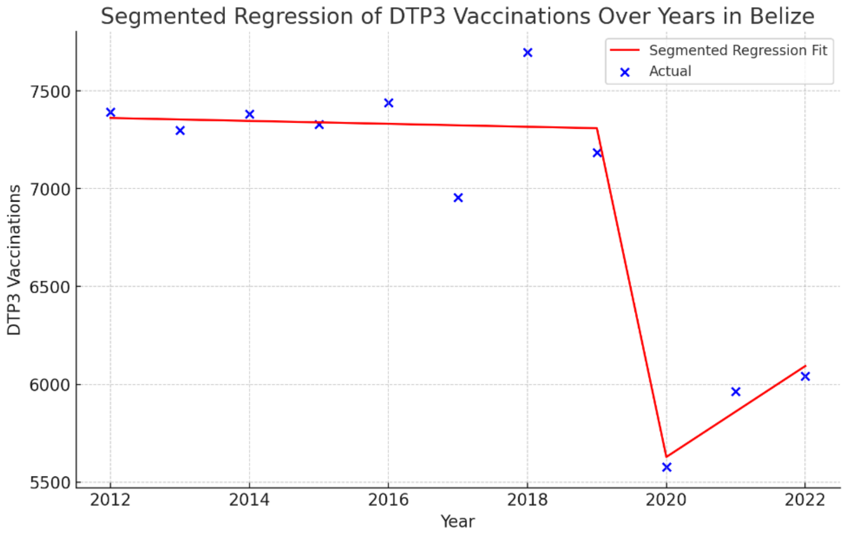

Figure S21. Segmented Regression Analysis of DTP3 Vaccination in Belize, representing the actual number of DTP3 vaccinations (in blue) and the fitted values from the segmented regression (in red).

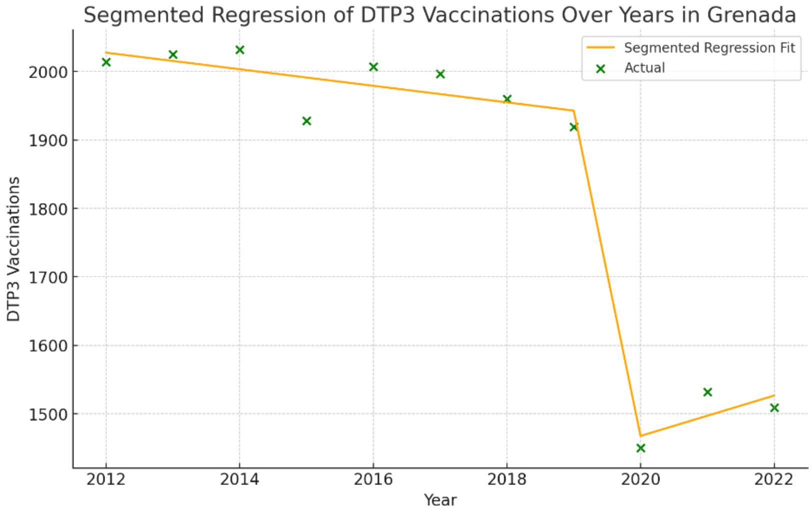

Figure S22 Segmented Regression Analysis of DTP3 vaccinations in Grenada represents the number of DTP3 vaccinations (in green) and the fitted values from the segmented regression (in orange)

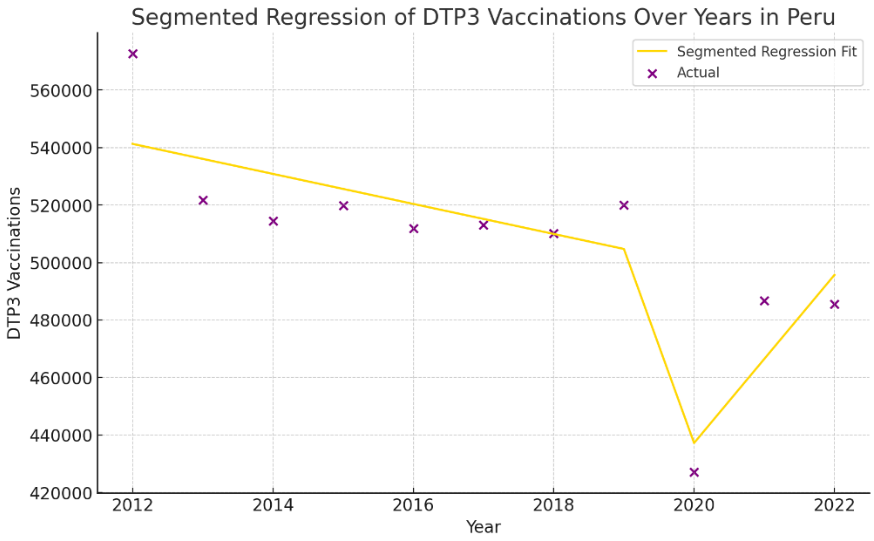

Figure S23. Segmented Regression Analysis of DTP3 vaccinations in Peru representing the actual DTP3 vaccination numbers (in purple) and the fitted values from the segmented regression (in gold).

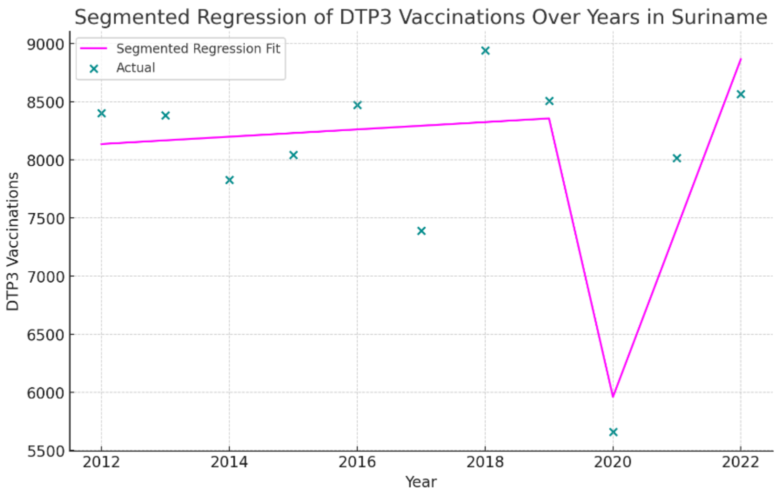

Figure S24. Segmented Regression Analysis of DTP3 vaccinations in Suriname representing the actual DTP3 vaccination numbers (in dark cyan) and the fitted values from the segmented regression (in magenta).

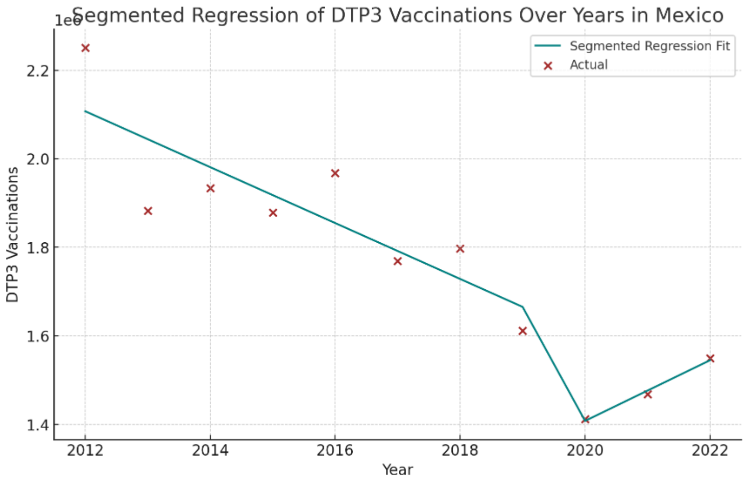

Figure S25 Segmented regression analysis of DTP3 vaccinations in Mexico representing the actual DTP3 vaccination numbers (in brown) and the predicted values from the segmented regression (in teal).

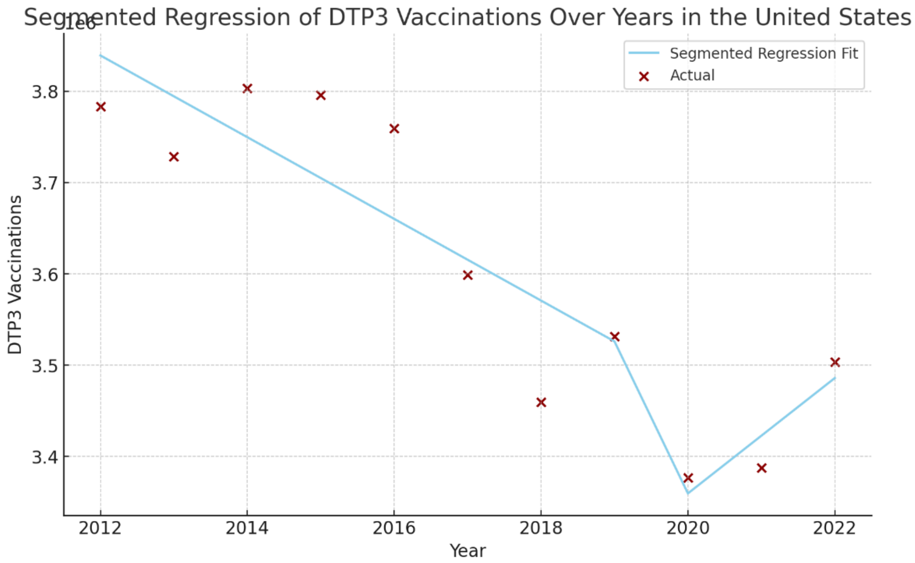

Figure S26. Segmented regression analysis of DTP3 vaccinations in the United States representing the actual DTP3 vaccination numbers in (dark red) against the predicted values from the segmented regression (in sky blue).
