# Supplementary material for: Has COVID-19 Affected DTP3 Vaccination in the Americas?
Source: Vaccines (Basel). 2024 Feb 25;12(3):238. doi: 10.3390/vaccines12030238 (PMC10975091; doi:10.3390/vaccines12030238)
Supplement: Supplementary file 1 [file vaccines-12-00238-s001.zip › Table S1.pdf]

Table S1. Summary Table Displaying Joinpoints close to 2020 and Chi-Square Test and Segmented Regression Analysis.

	Joinpoint			X^2	Segmented regression
**Country**	**Estimate**	**Lower 95%CI**	**Upper 95%CI**	**2019-22**	**2002-22**
Antigua and Barbuda	2017	2014	2020	<0.001	ns
Argentina				<0.001	ns
Bahamas	2020	2014	2020	ns	ns
Barbados				ns	ns
Belize	2019	2014	2020	0.003	<0.05
Bolivia (Plurinational State of)				<0.001	ns
Brazil				<0.001	ns
Canada	2018	2016	2020	ns	<0.05
Chile				ns	ns
Colombia	2019	2017	2020	<0.001	ns
Costa Rica	2017	2014	2020	ns	ns
Cuba				ns	ns
Dominica	2020	2014	2020	ns	ns
Dominican Republic				ns	ns
Ecuador	2018	2014	2020	<0.001	ns
El Salvador				<0.001	ns
Grenada	2018	2014	2020	ns	<0.05
Guatemala				<0.001	
Guyana				ns	ns
Haiti	2017	2014	2020	ns	ns
Honduras				<0.001	ns
Jamaica	2019	2014	2020	ns	ns
Mexico	2020	2014	2020	<0.001	ns
Nicaragua	2018	2016	2020	<0.001	ns
Panama				ns	ns
Paraguay	2018	2016	2019	<0.001	ns
Peru	2017	2014	2020	<0.001	<0.05
Saint Kitts and Nevis	2020	2014	2020	ns	ns
Saint Lucia	2019	2014	2020	ns	ns
Saint Vincent and the Grenadines	2017	2014	2020	ns	ns
Suriname	2020	2014	2020	ns	<0.05
Trinidad and Tobago				ns	ns
Uruguay	2020	2014	2020	ns	ns
United States				ns	ns
Venezuela				<0.001	ns

ns= No Significative
